# Resistance Switching and Memristive Hysteresis in Visible-Light-Activated Adsorbed ZnO Thin Films

**DOI:** 10.1038/s41598-018-20598-5

**Published:** 2018-02-01

**Authors:** Benjamin Kerr Barnes, Kausik S. Das

**Affiliations:** Department of Natural Sciences, University of Maryland Eastern Shore, Princess Anne, MD USA

## Abstract

The discovery of resistance switching memristors marks a paradigm shift in the search for alternative non-volatile memory components in the semiconductor industry. Normally a dielectric in these bistable memory cells changes its resistance with an applied electric field or current, albeit retaining the resistive state based on the history of the applied field. Despite showing immense potential, sustainable growth of this new memory technology is bogged down by several factors including cost, intricacies of design, lack of efficient tunability, and issues with scalability and eco-friendliness. Here, we demonstrate a simple arrangement wherein an ethanol-adsorbed ZnO thin film exhibits orders of magnitude change in resistance when activated by visible light. We show that there exists two stable ohmic states, one in the dark and the other in the illuminated regime, as well as a significant delay in the transition between these saturated states. We also demonstrate that visible light acts as a non-invasive tuning parameter for the bistable resistive states. Furthermore, a pinched hysteresis *I*-*V* response observed in these devices indicate what seems to be a new type of memristive behaviour.

## Introduction

Interest in materials exhibiting electrical resistance switching based on the history of applied voltage and current is in the upsurge, thanks to their numerous potential applications in designing new types of memory devices^1–5^, neuromorphic circuits and adaptive systems^6–11^. Although memristor, a name coined by Chua^12,13^, was theoretically predicted as the fourth, two port non-linear passive electrical component in the ’70s, it took nearly four decades to find the first real existence of this new circuit component and characterise it^14^. In these memristors electrical resistance depend on the history of applied voltage and charge flow and hence the current response to a voltage sweep is highly nonlinear and often characterized by a pinched hysteresis loop^15,16^. A large number of metal oxides, especially transition-metal oxides with perovskite structures including titanets, zirconets or magnetites as well as halides, chalcogenides and organic polymers have attracted special attention^17–23^, as suitable candidates for bistable switches.

Although resistance switching devices play a central role in many emerging technological fields mentioned above, in order for these technologies to be developed further and incorporated in the stream of consumer products, simple materials need to be found or synthesized and scalable manufacturing methods need to be developed. Advances in this area, so far, have not been significant due to a number of factors including environmental and economic considerations, and most importantly, engineering limitations. What we report here is an electronic device capable of resistance switching made from non-toxic, earth-abundant and environmentally friendly ZnO particles using a simple method which is conducive to mass production through roll printing. ZnO is well known for a myriad of other applications ranging from eco-friendly cosmetic sunscreens^24^ to solar cells^25^, field-effect transistors (FETs)^26^, light-emitting diodes (LEDs)^27^, ultra violet (UV) photo-detectors, low cost gas and bio-sensors^28–30^ to name a few. Zinc oxide’s wide band gap (~3.37 eV) and large exciton binding energy (~60 meV) make its electronic properties susceptible to electromagnetic radiation in the UV range (~368 nm). ZnO nanoparticles have recently been reported to show ethanol sensing properties also, where a dynamic change in electronic properties is postulated to be a function of the amount of ethanol gas present on the surface of nanoparticles in the dark^29^ or in the presence of a monochromatic UV light (wavelength ~370 nm)^31^. Surface adsorbed oxygen molecules in these ethanol sensors are believed to reduce the mobility of the free electron gas, thereby increasing overall resistance of the sample^32^. Thus, exposure to ethanol vapour in those ethanol sensors triggers displacement of the adsorbed oxygen molecules, in turn increasing the overall conductivity of the sample. The large adsorption energy of the surface-adsorbed oxygen ions (~0.35 eV) is responsible for their high thermal stability and the related difficulty to remove them from the ZnO surface at room temperature^33^. Because of this, metal oxide based ethanol sensors are believed to work better at significantly higher temperatures^31^. Plasma treatment on the otherhand is well known for surface modification of polymers and thin films for a plethora of applications including adhesion^34^, cleaning^35^, controlling oxygen vacancies^36^ and optical properties^37^ to name a few.

Recent studies of metal oxide based memristive switching includes a new ZnO microwire based UV mem-sensor fabricated using photocurable resin^30^ and a novel theoretical model of the memristive behaviour in ZnO nanowires based on the impedance variation of ZnO, triggered by surface interaction with metal adatoms^38^. Resistive hysteresis in flexible nanocomposites and colloidal suspensions was explained using Interfacial Coupling Mechanism (ICM) where the importance and effect of oxygen vacancies at the surface of ZnO fillers was highlighted^39^. A comprehensive review on ZnO thin films for memristive devices^40^ show that various thin film deposition techniques to design a memristor such as sputtering^41^, atomic layer deposition^42^ (ALD), pulsed layer deposition^43^ (PLD) and sol-gel bring different level of complexity, efficiency, cost effectiveness and bio-compatibility in each of the processes. However, majority of the memristors studied so far were designed in Metal-Insulator-Metal (MIM) configuration, where the ZnO thin film is sandwiched between a top and bottom metal electrodes. In these MIM configurations memristive switching is explained based on the formation and breakdown of continuous conductive filament growth^44–48^ between the two electrodes.

In this paper we present a novel experimental result wherein a thin film made from zinc oxide particles dispersed in ethanol exhibits orders of magnitude resistive switching induced by visible light at room temperature. Unlike memristors in MIM configuration, our ZnO thin film is deposited on a glass substrate using sol-gel method and the top surface is exposed to the ambient environment. Resistive switching in this configuration is observed irrespective of the size or morphology of ZnO particles, i.e., with ZnO powder, nanopowder or nanowires, when deposited on a plain uncoated glass substrate from a suspension in ethanol. Furthermore, a pinched hysteresis I-V response observed in these devices demonstrates what seems to be a new type of memristive behaviour. We trace the origin of this resistive switching behaviour to the surface characteristics of the thin films by using plasma-facilitated surface modifications, characterizing the surface modification by FTIR analysis and by measuring the switching behaviour as a function of the wave length of the incident light. We propose that this switching phenomenon arises from the displacement of adsorbed oxygen from the ZnO surface upon exposure to light.

## Results

### Resistance switching in dark and under illumination

**Figure 1:**
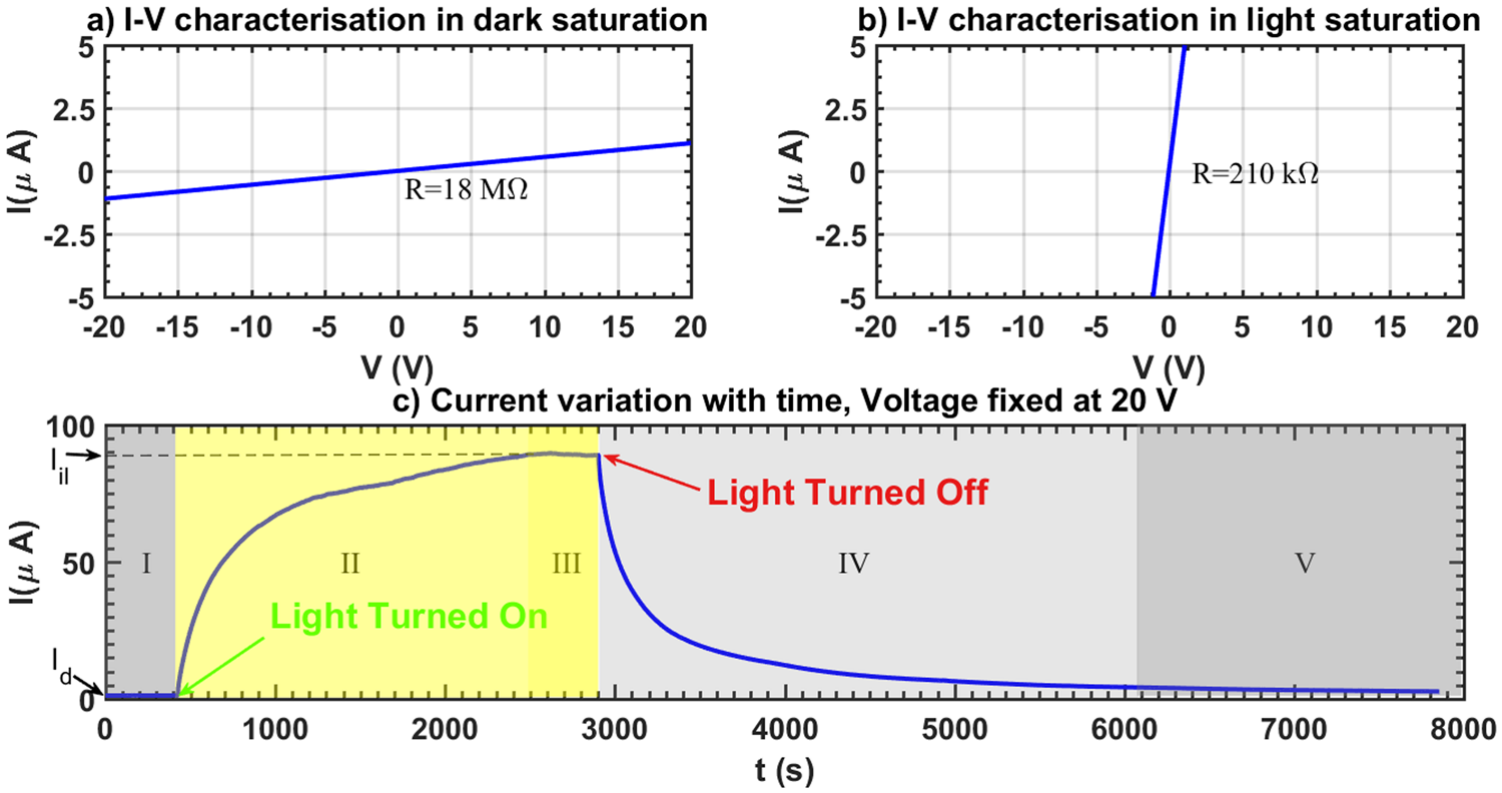
Resistance switching in a light-activated ethanol-adsorbed ZnO thin film. (**a**) The *I*-*V* characteristics in the dark shows a linear profile. The dark saturated resistance is calculated as the inverse of the slope of the linear profile. (**b**) Under illumination a similar linear *I*-*V* response is observed confirming the existence of bistable ohmic resistive states in the thin film. (**c**) Variation of current with time subjected to a constant 20 V bias is measured. Time evolution of current can be divided into five distinctive regimes. First one (I) represents dark saturated current *I*_d_ regime. In this state the thin film is in dark and the sample is in high resistive state (HRS). Once the solar simulator is turned on, as indicated in Fig. 1(c), current increases nonlinearly in this regime II until it reaches its second stable state, the low resistive state (LRS) in regime III. Once the current level reaches its asymptotic limit under illumination (*I*_d_), the light is turned off and the transition from the LRS to the HRS occurs in the IV^th^ regime. Finally current decays back to the saturated dark current level (*I*_d_) in regime V.

The adsorbed ZnO thin films undergo a visible-light-induced resistance switching between two stable ohmic states at ambient temperature. Although the transition time between the low and high resistive states depends on the size or morphology of ZnO particles, same characteristic switching behavior is observed in all thin films made of regular ZnO powder, nanopowder or nanowires. Light sensitive ZnO thin films are fabricated by depositing ZnO suspensions in ethanol on a glass substrate to form a sub-millimeter-thick coating (~0.70 mm). Electric contacts are made using a conductive glue on either end of the thin film. To record the current response to light the coated substrate is placed under a solar simulator. In the dark, i.e., even before the solar simulator is turned on an *I*-*V* sweep confirms linear Ohmic behaviour in this stable high resistive state (HRS) in the dark (Fig. 1a). Once the light is turned on, sufficiently long time of exposure to illumination brings the thin film to another stable, low resistive state (LRS) under illumination (Fig. 1b). The whole picture of resistance switching behaviour of the thin film under a constant 20 V potential difference is shown in Fig. 1c. The current response curve shows that the current increases nonlinearly with time from the stable HRS (regime I in Fig. 1c), in the dark to the stable LRS (regime III in Fig. 1c) under illumination. It requires a significant time interval (often of the order of ~10^3^ s) for the current to saturate, indicating a time dependent resistivity and a departure from ohmic behaviour in the transition from HRS to LRS. Sufficiently long time after the light is turned on (*t* → ∞), current reaches its illuminated asymptote marked by *I*_il_ in the regime III in Fig. 1c and then the light is turned off. Clearly the current doesn’t drop to the dark current level instantaneously, rather it behaves in a non-ohmic manner in this transition regime (regime IV in Fig. 1c). Eventually current decays back to the stable dark state (regime V in Fig. 1c), marked by the constant current *I*_*d*_ in the asymptotic limit. A proposed decay model for the current in the form

1 $$I={I}_{{\rm{d}}}+({I}_{{\rm{il}}}-{I}_{{\rm{d}}})\,\exp [-(t-{t}_{{\rm{off}}})/\tau ],$$

where *I*_il_ is the saturation current under illuminated condition, *I*_d_ is the dark saturation current, *t*_off_ is the time when the light is turned off and *τ* is a characteristic time constant, closely fits the experimental data, with a characteristic time constant *τ* ~ 400s, indicating an inductive behaviour similar to a RL circuit. The same time constant *τ* is used to fit the data for current growth model

2 $$I={I}_{{\rm{il}}}+({I}_{{\rm{d}}}-{I}_{{\rm{il}}})\,\exp [-(t-{t}_{{\rm{on}}})/\tau ],$$

**Figure 2:**
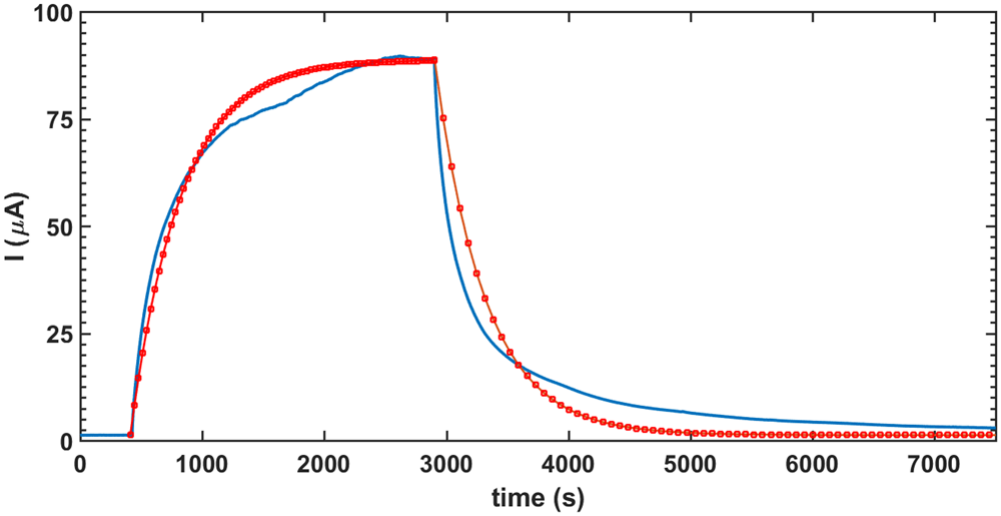
Experimental growth and decay of current (blue line) are compared with a proposed inductive growth and decay model (red dots). Growth of current is modelled as *I* = *I*_il_ + (*I*_d_ − *I*_il_) exp[−(*t* − *t*_on_)/*τ*], where *I*_il_ is the saturation current under illuminated condition, *I*_d_ is the dark saturation current, *t*_on_ is the time when the light is turned on and *τ* is a characteristic time constant. In this simulation *τ* is takes as 410 s. Once the light is turned off current decay is modelled as *I* = *I*_d_ + (*I*_il_ − *I*_d_) exp[−(*t* − *t*_off_)/*τ*], where *t*_off_ is the time when the light is turned off. Same *τ* is used for both the growth and the decay model. Blue line represents experimental data and the red square markers show the simulation results using the models given above. A slight dip in the current value near saturation is noted which may be attributed to the thermal effects of illumination on the sample.

**Figure 3:**
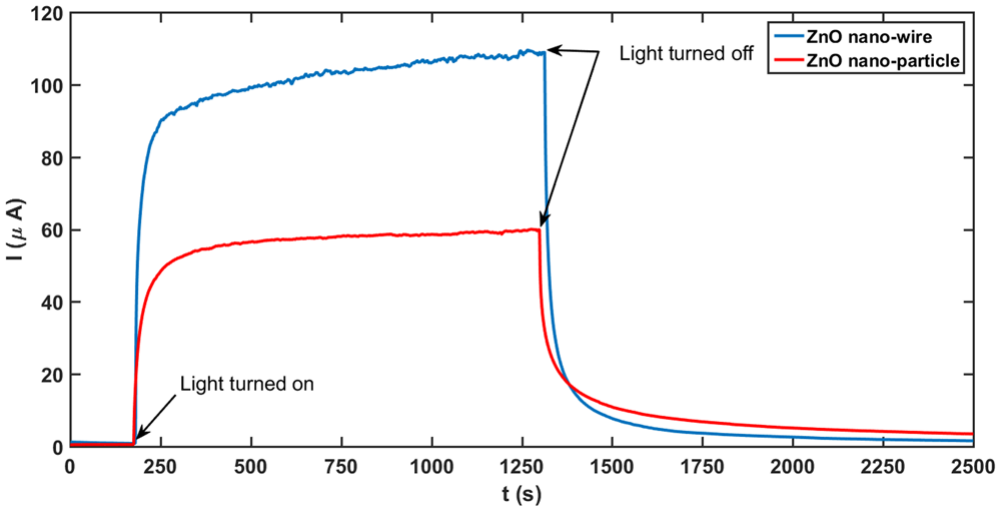
Growth and decay of current for ZnO nanowire and nanoparticle thin films under 20 V constant potential difference. Similar to the characteristics of non-nano thin films (Fig. 1), the nano-ZnO thin films also exhibit resistive switching in dark and under illuminated conditions.

*t*_on_ is the time when the light is turned on and is shown in the Fig. 2. We have also confirmed that this light activated resistance switching behaviour in ZnO is generic in nature and both ZnO nano and non-nano particles exhibit this behaviour. Growth and decay of output current for ZnO nanowires and nanoparticles are shown in Fig. 3, where nanowires exhibit larger resistance switching in comparison to nanoparticles and non-nanoparticles (Fig. 1). We hypothesise that the resistive switching occurs when electro-withdrawing molecules adsorbed to the ZnO surface are removed by incident light, thereby releasing their electrons into the conduction band. Powder ZnO and nanoparticle ZnO grains will tend to pack closely together in these thin films, limiting the available surface upon which this adsorption/desorption phenomenon can occur. However, when ZnO nanowires are used, a much higher surface area is exposed due to the less-uniform 3-D nature of this morphology. This means that many more electron-withdrawing molecules can be desorbed from nanowires than from other morphologies, resulting in a higher level of resistance switching.

### Frequency dependence and effect of surface modifications

**Figure 4:**
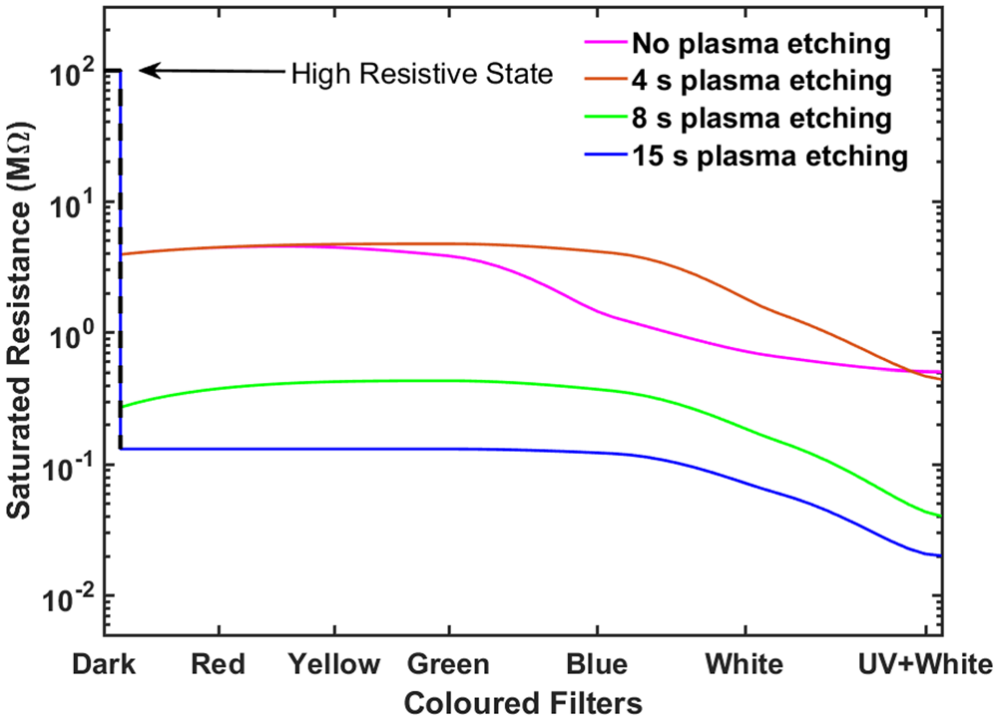
Photo-response of the ZnO thin films with different degrees of surface modifications under illumination. Before illumination the thin film was in the high resistive state (HRS). In the illuminated state (LRS) plasma etched by 4 s O_2_ plasma, the thin film shows minimal changes in the resistance with respect to the unetched one saturated under illuminated conditions. Increased plasma etching time reduces the variability of resistance to different colours, suggesting that the surface modification plays a significant role in the band gap adjustments. Clearly 15 s plasma-etched-thin-film has negligible preference to the incident light frequency on the low resistive state and the response to different colors of light is practically the same.

**Figure 5:**
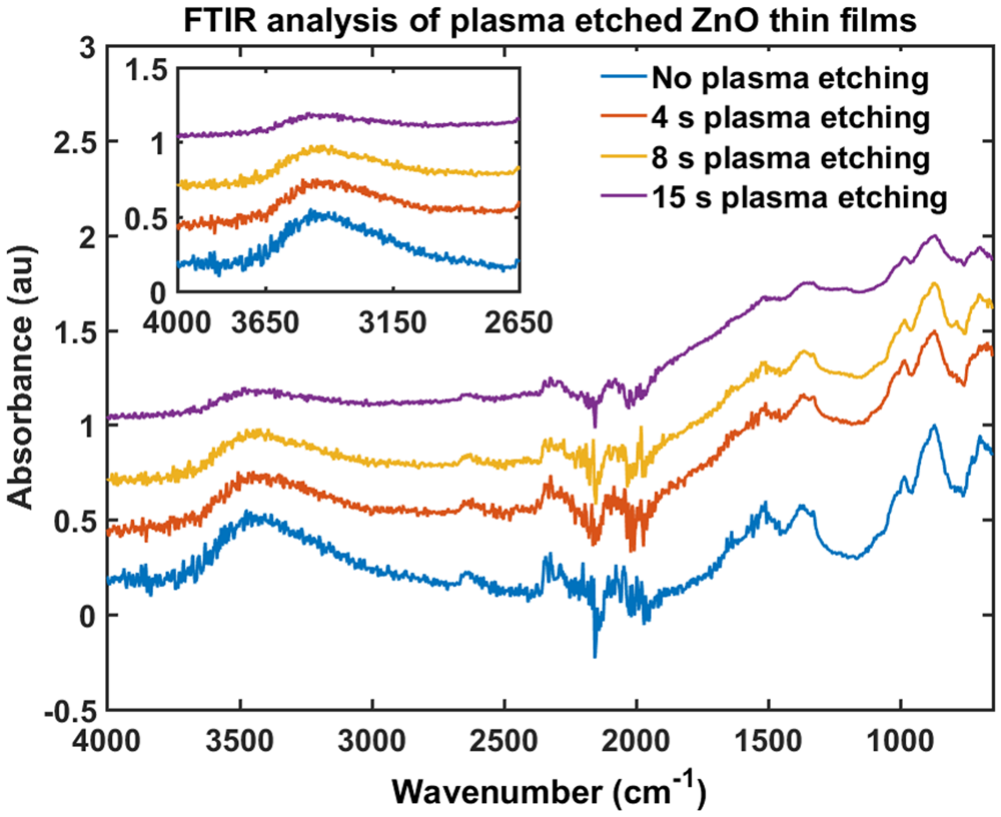
The figure shows the result of plasma etching a ZnO thin film. The film was fabricated as for all devices (deposition from ethanol suspension) followed by sintering. The etching was conducted for 4, 8 and 15 seconds. The inset showing the band at 3400 cm^−1^ due to the -OH bond in alcohols. The intensity of this signal clearly diminishes as the film is etched indicating a loss of electron-withdrawing groups from the surface of the material. The large signal at around 2100 cm^−1^ is from CO_2_ and the band at around 900 cm^−1^ is due to a small amount of glass particles that were scraped off of the device substrate. The graphs shown above are the FTIR analysis of ZnO nanowire thin films, however other ZnO thin films made of ZnO particles or nanoparticles also show similar behaviour under plasma etching.

**Figure 6:**
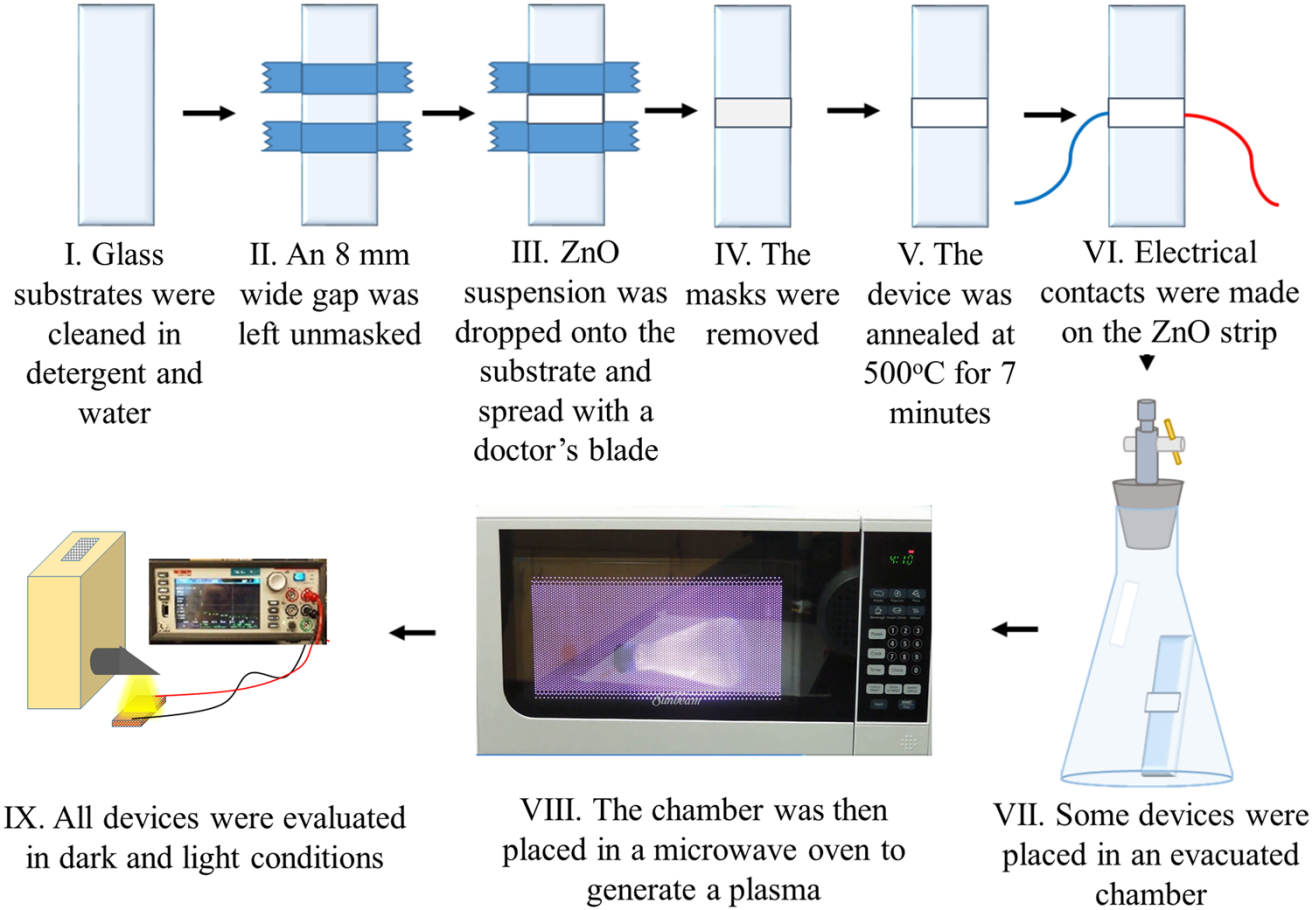
Fabrication of ethanol-adsorbed ZnO sensor devices. (**I**) A glass substrate is cleaned in ethanol and detergent and rinsed with deionized water. (**II**) Tape is applied to the substrate leaving an 8 mm wide space. (**III**) A few drops of ZnO suspension in ethanol, acetic acid, and water are placed in the gap between the tape and spread evenly with a razor blade. (**IV**) ZnO thin film is annealed at 500 °C. (**VI**) Two leads are attached to the ZnO strip with a minimal amount of silver-based conducting epoxy. (**VII**) Thin films to be plasma etched are placed in a vacuum flask which is then evacuated. (**VIII**) The flask containing the thin film is placed in a microwave oven and subjected to plasma for 4, 8, and 16 seconds. (**IX**) The current-voltage behavior of the sensors is evaluated under simulated sunlight at 0.5 Wm^−2^ intensity and in dark conditions (Fig. 1).

In the absence of UV components, evaluation of the effect of visible light of different wavelengths on the thin films are done by using UV and coloured Wratten filters in our experiments and the results are shown in Fig. 4. It is clear that even without the UV component, wavelengths in the visible range selected by the colour filters can facilitate the transition between the HRS and the LRS. It also shows that blue light (~600 nm) makes a higher impact on the transition process than other wavelengths. This result suggests a possible influence of adsorption of oxygen at the surface and the trapped ethanol in the bulk of the thin films on the band gap modification of ZnO nano and non-nanoparticles as observed in other materials such as surface adsorbed graphene or silicene^49,50^. The effect of surface modifications on the electrical response is also investigated by exposing the thin films, otherwise fabricated as above, to microwave-generated *O*_2_ plasma^51^. The light-saturated LRSs of the thin films etched for 4, 8, and 15 seconds as shown in Fig. 4 illustrates that the resistance of the illuminated state for all wavelengths decreases as the extent of surface modification is increased. This again points to a possible role of chemical species adsorbed on the ZnO surface in band gap modification. FTIR analysis before and after etching (Fig. 5) also supports the fact that exposure to the microwave oxygen plasma breaks down OH bonds at the surface of the thin films. A schematic of the whole plasma etching process is shown in the Fig. 6.

### Pinched hysteresis and memristive behaviour

As mentioned earlier that an investigation of the *I*-*V* characteristics of both the dark and the illuminated states confirms the existence of stable ohmic states with different resistivities. A voltage sweep generated by a source meter to characterise *I*-*V* behaviour in dark conditions reports a linear ohmic response (Fig. 1a) confirming proportional current variation with the applied voltage. The same process is repeated in the stable LRS under illuminated conditions to observe a different ohmic state (Fig. 1b), confirming the existence of visible-light-activated bistable resistive switching in ZnO thin films.

**Figure 7:**
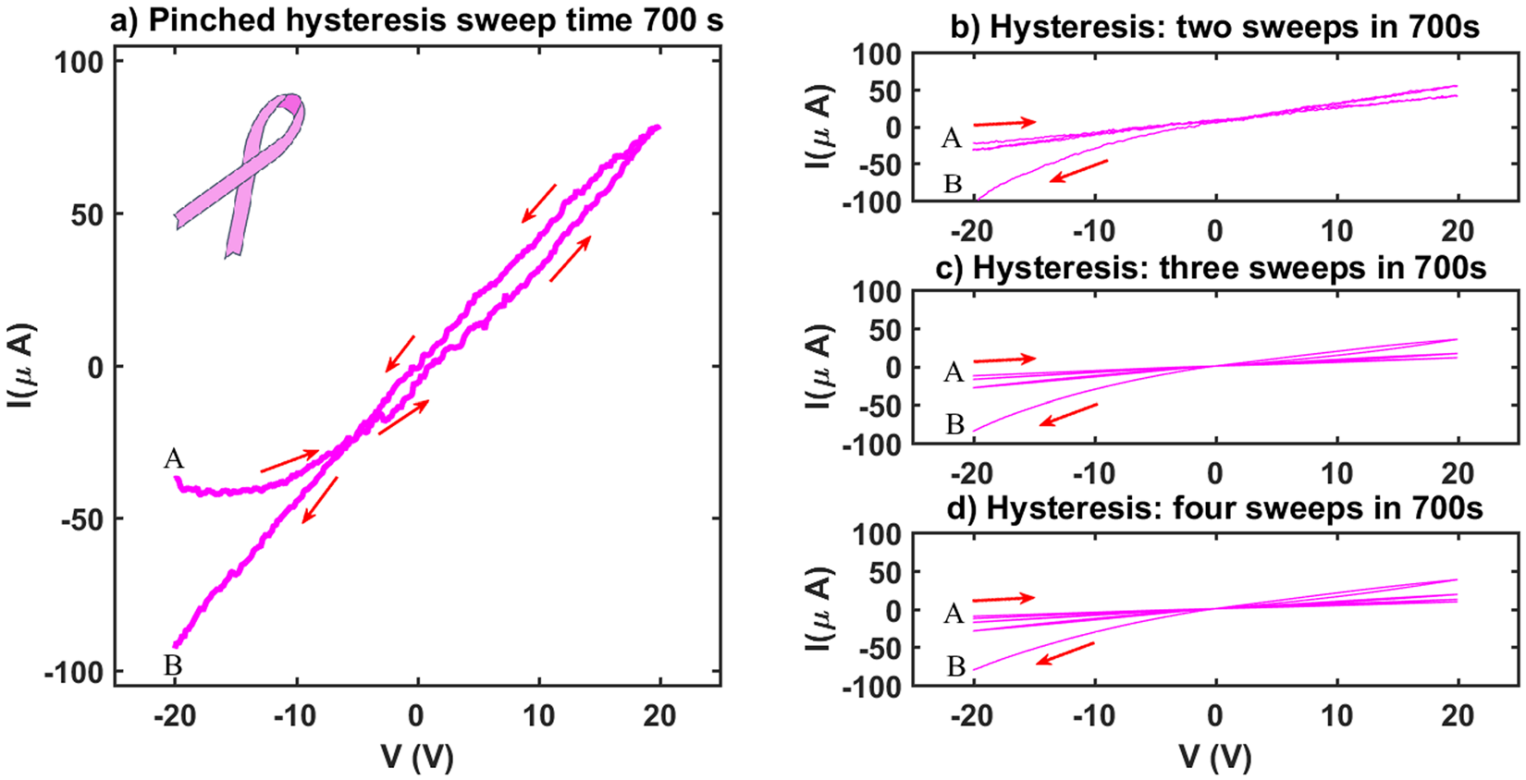
A Pink-Ribbon pinched hysteresis loop is observed. (**a**) Current versus voltage curve is measured at a frequency ~1.4 × 10^−3^ Hz of the voltage sweep. Light is turned on when the thin film is at its high resistance stable dark state (HRS) and the voltage difference between the end terminals starts at −20 V (point A). The potential difference is ramped up to 20 V in equal steps before eventually coming back to −20 V (point B). Light is still on at this end point B. If the light is turned off now, resistance slowly goes back to the high resistivity state (point A), a behaviour as described in Fig. 1c, in the IV^th^ regime. The overall *I*-*V* response curve visually resembles a ‘pink ribbon’, frequently used as a cancer awareness symbol. One important observation is that unlike most of the memristive pinching or crossover of *I*-*V* doesn’t occur at the origin (0, 0) of the *I*-*V* plane, clearly pointing out a memory effect on current response by the transient applied voltage. (**b**–**d**) *I*-*V* responses are measured at different sweep frequencies and highly nonlinear characteristics are observed.

However, *I*-*V* sweeps made in the transition regions (regions II and IV in Fig. 1c) reveal interesting non-ohmic hysteresis behavior, similar to what is normally observed in memristor devices^12,13^. Figure 7 illustrates this behavior for ZnO particles adsorbed in ethanol, although thin films made of ZnO nanoparticles and nanowires also show similar characteristic behaviors. The thin film is exposed to light at point **A** as voltage is ramped up from −20 V to 20 V and brought back to −20 V (at **B**) over the course of ~700 s. A pinched hysteresis loop, a typical fingerprint of memristive behaviours^52–54^, is observed. Although the resulting hysteresis loop clearly indicates a non-ohmic memristive behaviour unlike ideal memristors, pinching in this case doesn’t occur at the origin (0, 0) of the *I*-*V* plane. In this transition region current depends on the instantaneous voltage and the corresponding resistance, ensuring the thin films to behave according to the history of the resistive states in its trajectory. Noticing a striking visual similarity between the shape of the loop and the cancer awareness pink ribbon symbol, we call this behaviour ‘pink-ribbon-memristive’ behaviour of ethanol-adsorbed ZnO thin films. We hypothesize that this memristive behaviour is an artifact of the change in electron mobility in the conduction band of the adsorbed ZnO thin films in the transition regime. Ethanol molecules trapped in the bulk ZnO and oxygen molecules adsorbed at the surface play critical roles in the transition from HRS to LRS and the overall history dependent resistive dynamics. In dark state (HRS) oxygen molecules, weakly adsorbed at the surface, trap electrons from the conduction band of ZnO reducing the mobility of electron gas under an applied potential difference. This may be seen as equivalent to introducing effective viscosity and creation of a viscous boundary layer in the conduction band, in the Drude model of free electron gas flow^55^. At **A** (Fig. 7a) as light is turned on photo excited oxygen molecules start to desorb from the surface, in turn freeing up electrons and gradual restoration of free electron mobility in the conduction band of ZnO. Two time scales are at play here: a) the time scale related to the light activated desorption kinetics of oxygen molecules from the surface and b) electron diffusion time scale in the conduction band. Overall transition from the **A** to **B** largely depends on the interplay between these two time scales and hence we observe the characteristic nonlinear behaviour in the *I*-*V* curve. Similar to the role played by the filamental growth and decay in normal memory dependent resistive dynamics, here the growth (in dark) and decay (under illumination) of the electrical double layer at the surface created by the trapping and release of the conduction band electrons by the adsorbed oxygen molecules determine the ‘pink-ribbon-memristive’ hysteresis. Because of this time and history dependence of the electrical resistivity, the current at **A**, when the resistance was high and the light is turned on is different from that at **B** where the resistance of the thin film is different because of the immediate past resistive history of the thin film. It is also observed that unlike filamental growth dependent memristors, where a sharp transition between LRS and HRS is observed whenever filament is broken, here the transition from **A** to **B** when the light is turned off after the end of the sweep is slow and takes hundreds of seconds because of the slow readsorption kinetics of the ambient oxygen molecules at the surface and subsequent retrapping of conduction electrons. This behaviour is observed in every thin films made from ZnO particles, nanoparticles or nanowires irrespective of size or morphology.

**Figure 8:**
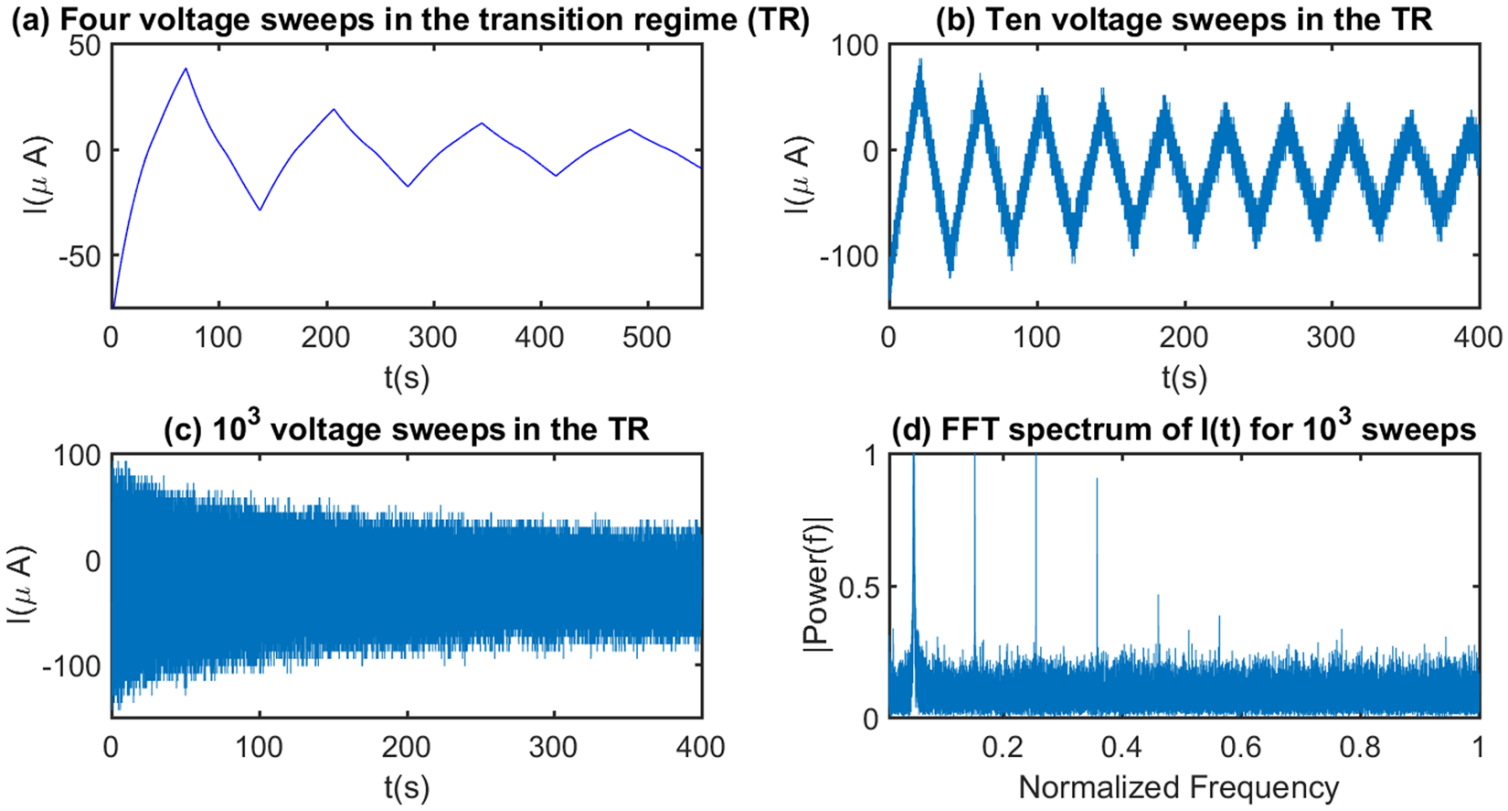
Time series data of the output current plotted for different voltage sweep frequencies. Input voltage is varied between −20 V to +20 V at different time periods. (**a**) Four sweeps are made in the voltage in the transition regime, (**b**) ten weeps are made in the transition regime and shorter frequencies start to perturb the input frequency. (**c**) Input voltage makes thousand sweeps and the output current shows the full spectrum of frequencies present in the time series. This highly nonlinear behaviour points to the possible transition to chaos. (**d**) FFT analysis of the corresponding time series of thousand sweeps show presence of all possible output frequencies generated in response to the fixed frequency input voltage sweep.

A complex response to multiple voltage sweeps with smaller sweeping times in the same transition regime (IV of Fig. 1c) is shown in Fig. 7(a–c). In response to the increasing voltage sweep frequency the time series of the output current shows generation of shorter time scales in the output. The Fast Fourier Transform (FFT) of the time series data of large frequency sweeps (such as Fig. 7c) shows a noise like highly nonlinear frequency masking of the dominant input frequency. This observation points to a possible transition to chaos in large sweep frequency regime and the corresponding time series in Fig. 8 is highly representative of that indication. A ‘strange-attractor’ type behaviour is also observed in the *I*-*V* plane of the corresponding *I*-*V* loops which may be used to design a single component Chua circuit^56^ for generating chaos and related applications.

## Discussion

In conclusion, we have demonstrated a novel, visible-light-activated, resistive switching phenomenon in nano (Fig. 3) and non-nano (Fig. 1) ZnO thin films with surface and bulk adsorbed ethanol at room temperatures. The switching between the LRS and the HRS bears the signature of an inductive delay. A pink-ribbon *I*-*V* curve in the transition regime shows a pinched hysteresis behaviour similar to that of a memristor. We hypothesise that in presence of trapped ethanol in the bulk of the thin film oxygen adsorption and desorption on the ZnO thin film surface plays a major role in this bistable switching behaviour^32,33^. Due to the maximum adhesion between ethanol and zinc oxide particles deposited from suspension, the zinc oxide thin films get heavily saturated with tightly-bonded ethanol both in the bulk and at the surface. Some ethanol molecules at the surface is converted to dangling OH groups, some more is decomposed to hydrogen and ethylene upon annealing at high temperature (~500 °C)^33,57^. However, a substantial amount of ethanol remains associated with the material bulk, and remains trapped inside the thin film due to the tight packing of the ZnO particles. The hydrogen which evolves during decomposition at the surface remains in close contact with the ZnO particles due to the presence of trapped ethanol and fills some oxygen vacancies^58–60^. As the substrate cools down components of the ambient atmosphere such as molecular oxygen as well as water molecules weakly adsorb to the ZnO surface. We propose that the n-type nature of ZnO^61^ naturally attracts oxygen molecules^32^ and the electronegative nature of the adsorbed oxygen attracts the ZnO electrons from its conduction band subsequently creating an electrical double layer (EDL) and a *ζ* potential between the adsorbed electronegative oxygen and the attracted electrons across the interface. This *ζ* potential in the transverse direction to the motion of the charge carriers interacts with their mobility of electrons in the thin film, in turn, reducing its conductivity in dark.

Upon exposure to light, the weakly adsorbed atmospheric oxygen molecules are desorbed, thereby releasing the ZnO electrons back into the ZnO conduction band. This increase in the number of shallow donors in the conduction band leads to enhanced photoconductivity^59,62^ of the sample. The switching is still due to the photo-desorption of surface adsorbed atmospheric molecules^63,64^, but the ethanol trapped in the bulk enhances the effect by increasing total number of charge carriers. The delay observed in this transition is a result of the desorption kinetics of the adsorbed gases. When exposed to light for a time period of sufficient length, the complete surface desorption of these gases leads to the asymptotically stable low resistive state which remains constant until the light is turned off again. Turning off the light also allows atmospheric gases, especially oxygen to re-adsorb to the ZnO surface and subsequently electrical resistance to increase. The delay in resistance switching observed in this process is again the result of the kinetics of re-adsorption.

**Figure 9:**
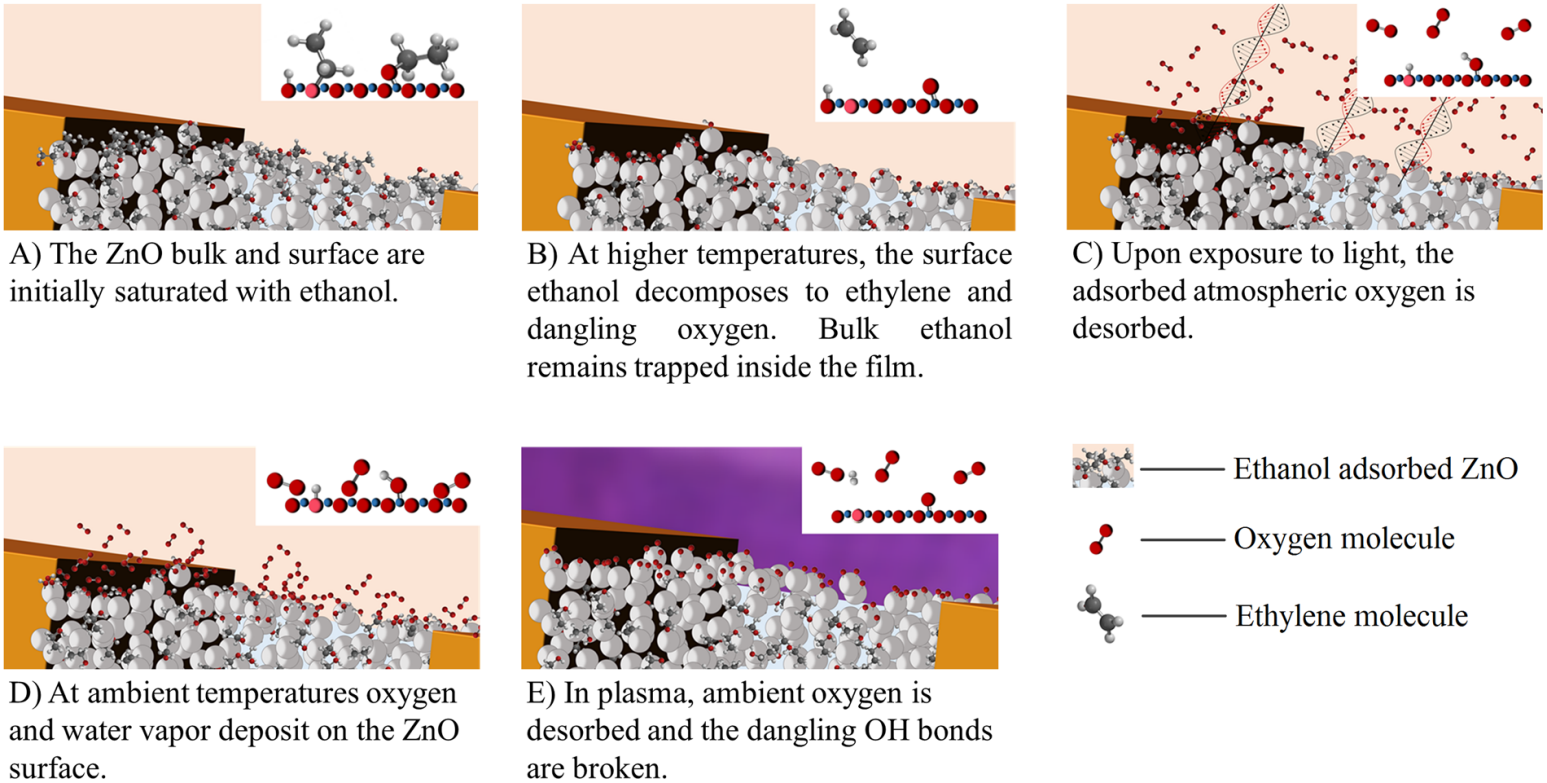
Proposed reaction pathway for the resistance switching mechanism. (**A**) Thin film made from ZnO particles suspended in ethanol. The inset shows surface adsorbed ethanol, whereas in the majority of the ethanol molecules are trapped in the tightly packed ZnO particles in the bulk. (**B**) At higher temperatures, the surface ethanol decomposes to ethylene and dangling oxygen. Bulk ethanol mostly remains trapped inside the film. (**C**) Exposure to incident photons removes the weakly connected oxygen, thereby releasing electrons back to the conduction band of ZnO. (**D**) Once the light is turned off, atmospheric oxygen and water molecules readsorb at the active cites. (**E**) Plasma treatment of the sample breaks down the dangling OH bonds.

Plasma etching breaks the O-H bonds of the residual surface OH groups which formerly were withdrawing electrons. This generates a proton, which leaves the ZnO surface, and a dangling oxygen with an associated electron at the interface. The extra electrons in the thin film further enhances the photoconductivity by increasing the photoresponse and recovery rates as well as by reducing the resistance in both lighted and dark conditions. Pre and post etching FTIR analysis (Fig. 5) and also confirms breakdown of -OH groups from the ZnO thin film surface due to the plasma treatment. A schematic of the proposed reaction pathway is given in Fig. 9.

## Methods

ZnO thin films are fabricated by depositing an 8 mm wide strip of zinc oxide (powder from JT Baker, nanopowder, and nanowires from Sigma Aldrich) suspension in acetic acid (1% v/v) and ethanol (67% v/v) (both from Fisher Scientific) in water on a glass substrate using the doctor’s blade method. Commercial ZnO is used in this study, but it can be easily synthesized by a wide variety of traditional methods such sol-gel, solvothermal, and hydrothermal techniques^65^, as well as by emerging green synthesis routes which utilize stabilizers extracted from algae, plants, and bacteria^66^. The substrate coated with the ZnO film is then annealed in air by ramping the temperature from room temperature to 500 °C over 7 minutes. Thickness of the ZnO thin films are determined by a Laser displacement meter (Keyence, LK-H022) to be ~0.7 mm. Thin copper leads are then connected to each end of the zinc oxide strip with a minimal amount of silver epoxy glue manufactured by Atom Adhesives (electrical resistivity 0.0001 Ω cm). To evaluate the correlation between the surface conditions and effective resistivity of the thin films we have used microwave generated *O*_2_ plasma to etch the thin film surface with different degrees of plasma treatment and recorded the response to light for each of the sensors. Microwave plasma is generated in the following way (Fig. 6)^51^: ZnO coated glass slides are first placed in a vacuum flask. Using a vacuum pump, the flask is evacuated down to a pressure less than 6 Torr and then placed in a microwave. The microwave is run for 4, 8 or 15 seconds while deep purple plasma visible through the microwave window. The electrical properties of the ZnO films were determined by attaching the copper leads at the opposite ends of the ZnO strip with a Kiethely 2450 SourceMeter via test probe hook clips, allowing for great precision and stability across a range of voltage and frequency parameters. Current as a function of time was measured while applying a constant potential of 20 V in the dark, and after the device was exposed to light of 1 Sun intensity. The IV behaviour of the material was also elucidated using the SourceMeter in each of the dark saturation, transition, and light saturation regimes at a range of frequencies. Voltage sweeps between −20 V and +20 V (Figs 7 and 8) were generated and the corresponding current and the time series were also measured.

## References

[1] Jo SH, Lu W (2008). Cmos compatible nanoscale nonvolatile resistance switching memory. Nano letters.

[2] Kwon D-H (2010). Atomic structure of conducting nanofilaments in tio2 resistive switching memory. Nat. nanotechnology.

[3] Park WY (2010). A pt/tio2/ti schottky-type selection diode for alleviating the sneak current in resistance switching memory arrays. Nanotechnol..

[4] Seo S (2004). Reproducible resistance switching in polycrystalline nio films. Appl. Phys. Lett..

[5] Lai Y-S, Tu C-H, Kwong D-L, Chen J-S (2005). Bistable resistance switching of poly (n-vinylcarbazole) films for nonvolatile memory applications. Appl. Phys. Lett..

[6] Jo SH (2010). Nanoscale memristor device as synapse in neuromorphic systems. Nano letters.

[7] Yu S, Wu Y, Jeyasingh R, Kuzum D, Wong H-SP (2011). An electronic synapse device based on metal oxide resistive switching memory for neuromorphic computation. IEEE Transactions on Electron Devices.

[8] Kim K-H (2011). A functional hybrid memristor crossbar-array/cmos system for data storage and neuromorphic applications. Nano letters.

[9] Indiveri G, Linares-Barranco B, Legenstein R, Deligeorgis G, Prodromakis T (2013). Integration of nanoscale memristor synapses in neuromorphic computing architectures. Nanotechnol..

[10] Driscoll T (2010). Memristive adaptive filters. Appl. Phys. Lett..

[11] Linares-Barranco, B. & Serrano-Gotarredona, T. Exploiting memristance in adaptive asynchronous spiking neuromorphic nanotechnology systems. In *Nanotechnology*, *2009*. *IEEE*-*NANO 2009*. *9th IEEE Conference on*, 601–604 (IEEE, 2009).

[12] Chua L (1971). Memristor-the missing circuit element. IEEE Transactions on circuit theory.

[13] Chua LO, Kang SM (1976). Memristive devices and systems. Proc. IEEE.

[14] Strukov DB, Snider GS, Stewart DR, Williams RS (2008). The missing memristor found. Nat.

[15] Chanthbouala A (2012). A ferroelectric memristor. Nat. materials.

[16] Mazumder P, Kang S-M, Waser R (2012). Memristors: devices, models, and applications. Proc. IEEE.

[17] Waser R, Aono M (2007). Nanoionics-based resistive switching memories. Nat. materials.

[18] Lee M-J (2009). Electrical manipulation of nanofilaments in transition-metal oxides for resistance-based memory. Nano letters.

[19] Ahn S-E (2008). Write current reduction in transition metal oxide based resistance change memory. Adv. materials.

[20] Rossel C, Meijer G, Bremaud D, Widmer D (2001). Electrical current distribution across a metal–insulator–metal structure during bistable switching. J. Appl. Phys..

[21] Chen X, Wu N, Strozier J, Ignatiev A (2006). Spatially extended nature of resistive switching in perovskite oxide thin films. Appl. physics letters.

[22] Redaelli A (2004). Electronic switching effect and phase-change transition in chalcogenide materials. IEEE Electron Device Lett..

[23] Jang J, Pan F, Braam K, Subramanian V (2012). Resistance switching characteristics of solid electrolyte chalcogenide ag2se nanoparticles for flexible nonvolatile memory applications. Adv. Mater..

[24] Cross SE (2007). Human skin penetration of sunscreen nanoparticles: in-vitro assessment of a novel micronized zinc oxide formulation. Ski. pharmacology physiology.

[25] Law M, Greene LE, Johnson JC, Saykally R, Yang P (2005). Nanowire dye-sensitized solar cells. Nat. materials.

[26] Fan Z, Wang D, Chang P-C, Tseng W-Y, Lu JG (2004). Zno nanowire field-effect transistor and oxygen sensing property. Appl. Phys. Lett..

[27] Lim J-H (2006). Uv electroluminescence emission from zno light-emitting diodes grown by high-temperature radiofrequency sputtering. Adv. Mater..

[28] Guo J (2014). High-performance gas sensor based on zno nanowires functionalized by au nanoparticles. Sensors Actuators B: Chem..

[29] Jing Z, Zhan J (2008). Fabrication and gas-sensing properties of porous zno nanoplates. Adv. Mater..

[30] Chiolerio A (2015). Ultraviolet mem-sensors: flexible anisotropic composites featuring giant photocurrent enhancement. Nano Res..

[31] Zheng, Z., Yao, J., Wang, B. & Yang, G. Light-controlling, flexible and transparent ethanol gas sensor based on zno nanoparticles for wearable devices. *Sci*. *reports* **5** (2015). 10.1038/srep11070 PMC4468465 26076705

[32] Korir K, Catellani A, Cicero G (2014). Ethanol gas sensing mechanism in zno nanowires: an ab initio study. The J. Phys. Chem. C.

[33] Kwak G, Yong K (2008). Adsorption and reaction of ethanol on zno nanowires. The J. Phys. Chem. C.

[34] Borcia C, Punga I, Borcia G (2014). Surface properties and hydrophobic recovery of polymers treated by atmospheric-pressure plasma. Appl. Surf. Sci..

[35] Aronsson B-O, Lausmaa J, Kasemo B (1997). Glow discharge plasma treatment for surface cleaning and modification of metallic biomaterials. J. Biomed. Mater. Res. Part A.

[36] Xu L (2016). Plasma-engraved co3o4 nanosheets with oxygen vacancies and high surface area for the oxygen evolution reaction. Angewandte Chemie.

[37] Gokus T (2009). Making graphene luminescent by oxygen plasma treatment. ACS nano.

[38] Raffone F, Risplendi F, Cicero G (2016). A new theoretical insight into zno nws memristive behavior. Nano letters.

[39] Chiolerio A, Roppolo I, Bejtka K, Asvarov A, Pirri C (2016). Resistive hysteresis in flexible nanocomposites and colloidal suspensions: interfacial coupling mechanism unveiled. RSC Adv..

[40] Laurenti M, Porro S, Pirri CF, Ricciardi C, Chiolerio A (2017). Zinc oxide thin films for memristive devices: a review. Critical Rev. Solid State Mater. Sci..

[41] Zoolfakar AS (2013). Engineering electrodeposited zno films and their memristive switching performance. Phys. Chem. Chem. Phys..

[42] Mundle R, Carvajal C, Pradhan AK (2016). Zno/al: Zno transparent resistive switching devices grown by atomic layer deposition for memristor applications. Langmuir.

[43] Macaluso R (2014). Resistive switching behaviour in zno and vo2 memristors grown by pulsed laser deposition. Electron. Lett..

[44] Huang C-H (2012). Zno1–x nanorod arrays/zno thin film bilayer structure: from homojunction diode and high-performance memristor to complementary 1d1r application. Acs Nano.

[45] Sun Y (2015). High on–off ratio improvement of zno-based forming-free memristor by surface hydrogen annealing. ACS applied materials & interfaces.

[46] Dongale TD (2015). Development of ag/zno/fto thin film memristor using aqueous chemical route. Mater. Sci. Semicond. Process..

[47] Sun Y (2016). Influence of carrier concentration on the resistive switching characteristics of a zno-based memristor. Nano Res..

[48] Kumar A, Baghini M (2014). Experimental study for selection of electrode material for zno-based memristors. Electron. Lett..

[49] Balog R (2010). Bandgap opening in graphene induced by patterned hydrogen adsorption. Nat. materials.

[50] Quhe R (2012). Tunable and sizable band gap in silicene by surface adsorption. Sci. reports.

[51] Lo, K., Summers, J., Bulovic, V. & Ram, R. Nanomaker. massachusetts institute of technology: Mit opencourseware. https://ocw.mit.edu **6**.**S079** (Spring 2013).

[52] Szot K, Speier W, Bihlmayer G, Waser R (2006). Switching the electrical resistance of individual dislocations in single- crystalline srtio3. Nat. materials.

[53] Bessonov AA (2015). Layered memristive and memcapacitive switches for printable electronics. Nat. materials.

[54] Maeda K (2005). Gan: Zno solid solution as a photocatalyst for visible-light-driven overall water splitting. J. Am. Chem. Soc..

[55] Steinberg M (1958). Viscosity of the electron gas in metals. Phys. Rev..

[56] Chua LO, Kocarev L, Eckert K, Itoh M (1992). Experimental chaos synchronization in chua’s circuit. Int. J. Bifurc. Chaos.

[57] Nagao M, Morimoto T (1980). Adsorption of alcohols on zinc oxide surfaces. The J. Phys. Chem..

[58] Jokela S, McCluskey M (2005). Structure and stability of o-h donors in zno from high-pressure and infrared spectroscopy. Phys. Rev. B.

[59] Van de Walle CG (2000). Hydrogen as a cause of doping in zinc oxide. Phys. review letters.

[60] Hofmann DM (2002). Hydrogen: a relevant shallow donor in zinc oxide. Phys. Rev. Lett..

[61] Shim M, Guyot-Sionnest P (2000). N-type colloidal semiconductor nanocrystals. Nat..

[62] Nahm, H.-H., Park, C. & Kim, Y.-S. Bistability of hydrogen in zno: Origin of doping limit and persistent photoconductivity. *Sci*. *reports* **4** (2014). 10.1038/srep04124 PMC3927214 24535157

[63] Cammi, D. & Ronning, C. Persistent photoconductivity in zno nanowires in different atmospheres. *Adv*. *Condens*. *Matter Phys*. **2014** (2014).

[64] Madel M (2017). Persistent photoconductivity in zno nanowires: Influence of oxygen and argon ambient. J. Appl. Phys..

[65] Kołodziejczak-Radzimska A, Jesionowski T (2014). Zinc oxide—from synthesis to application: a review. Mater..

[66] Mirzaei H, Darroudi M (2017). Zinc oxide nanoparticles: biological synthesis and biomedical applications. Ceram. Int..

